# In Silico Screening of IL-1β Production Inhibitors Using Chemometric Tools

**Published:** 2017

**Authors:** Amirhossein Sakhteman, Najmeh Edraki, Bahram Hemmateenejad, Ramin Miri, Alireza Foroumadi, Abbas Shafiee, Mehdi Khoshneviszadeh

**Affiliations:** a *Department of Medicinal Chemistry, Faculty of Pharmacy, Shiraz University of Medical Sciences, Shiraz, Iran.*; b *Medicinal and Natural Products Chemistry Research Center, Shiraz University of Medical Sciences, Shiraz, Iran.*; c *Department of Chemistry, Shiraz University, Shiraz, 71454, Iran.*; d *Pharmaceutical Sciences Research Center, Tehran University of Medical Sciences, Tehran, Iran.*; e *Department of Medicinal Chemistry, Faculty of Pharmacy, Tehran University of Medical Sciences, Tehran, Iran.*

**Keywords:** QSAR, IL-1β, Pyridazine, *in silico* screening, MLR

## Abstract

The IL-1β plays a major role in inflammatory disorders and IL-1β production inhibitors can be used in the treatment of inflammatory and related diseases. In this study, quantitative relationships between the structures of 46 pyridazine derivatives (inhibitors of IL-1β production) and their activities were investigated by Multiple Linear Regression (MLR) technique Stepwise Regression Method (ES-SWR). The genetic algorithm (GA) has been proposed for improvement of the performance of the MLR modeling by choosing the most relevant descriptors. The results show that eight descriptors are able to describe about 83.70% of the variance in the experimental activity of the molecules in the training set. The physical meaning of the selected descriptors is discussed in detail. Power predictions of the QSAR models developed were evaluated using cross-validation, and validation through an external prediction set. The results showed satisfactory goodness-of-fit, robustness and perfect external predictive performance. The applicability domain was used to define the area of reliable predictions. Furthermore, the *in silico *screening technique was applied in order to predict the structure and potency of new compounds of this type using the proposed QSAR model.

## Introduction

In recent years, many efforts have been done toward the development of new therapeutic agents in the area of inflammatory diseases. Cytokines are multifunctional proteins that responsible for host defense mechanisms such as inflammatory, immune and hematogenic responses (1). Cytokines have been categorized as being proinflammatory (IL-1β, TNF-α, IL-6...) or anti-inflammatory (TGF-β, IL-10, IL-13...) depending on their effects on the immune system. Inflammatory cytokines play an important role in inflammatory disease. Consequently, inhibition the production of IL-1β can reduce levels of these proinflammatory cytokine, and thereby reduce inflammation and prevent destruction effects in diseases such as rheumatoid arthritis (RA), osteoarthritis (OA) and Crohn’s disease (2). For the rational design of novel IL-1β production inhibitors, quantitative structure-activity relationships (QSAR) models and *in silico* screening could be useful (3). QSAR is a powerful tool to study the relationship between activity and structural parameters and could be used for design new biological active compounds and predict their potency, toxicity, ADME,etc. In this study, a series of 5, 6-bis (4-methoxyphenyl) -pyridazin derivatives with IL-1β production inhibitory activity discovered by Matsuda *et al* (4, 5). According to our literatures review, no QSAR study was carried out on the mentioned series. In the present investigation, a quantitative structure–activity relationship was explored using different molecular descriptors. Previous studies demonstrated that linear QSAR models had good predictive ability therefore (6-9), a virtual screening study was then carried out to identify novel biologically active patterns by modification of the original molecules. The study led to the identification of novel structures, which are potent IL-1β production inhibitors based on the QSAR model. The structures were filtered using the domain of applicability of the QSAR model. 

## Materials and methods

*Data set*

The biological data used in this study are the IL-1β production inhibitory (IC_50_) activity of a series of 5,6-bis (4-methoxyphenyl)-pyridazin derivatives (4, 5). The structural features and biological activity of these compounds are listed in Table 1. The biological data were converted to logarithmic scale (pIC_50_) and then used for subsequent QSAR analysis as dependent variables. The original data set was divided into a training set (n = 35) and a prediction set (n = 11) using the popular Kennard and Stones algorithm (10, 11). This algorithm has been widespread used with great success in many QSAR studies (12-14).

*Descriptor generation*

The chemical structure of molecules was constructed using Hyperchem package (Version 7, Hypercube Inc., Florida 32601 USA). The Z-matrices of the structures were provided by the software and transferred to the Gaussian 98 program. Complete geometry optimization was performed taking the most extended conformations as starting geometries. Semi empirical molecular orbital calculations (AM1) of the structures were performed using Gaussian 98 program. A large numbers of molecular descriptors were calculated using HyperChem, Gaussian 98 and Dragon Packages. Gaussian 98 was employed for calculation of different quantum chemical descriptors including dipole moment (DM), local charges, HOMO and LUMO energies, hardness (g); softness (S); electronegativity (v); and electrophilicity (x). Dragon software calculated different functional groups, topological, geometrical and constitutional descriptors for each molecule, and some chemical parameters including molecular volume (V), molecular surface area (SA), hydrophobicity (log P), hydration energy (HE) were calculated using Hyperchem software.

*Data processing and modeling*

The calculated descriptors were collected in a data matrix, D. First the descriptors were checked for constant or near-constant values and those detected were removed from the original data matrix. Then, the correlation of descriptors with each other’s and with the activity data was determined. Among the collinear descriptors detected (r > 0.9), one of them that had the highest correlation with activity was retained, and the rest were omitted (15, 16). For the development of QSAR equation stepwise multiple linear regression (MLR) was used. In stepwise regression, a multiple-term linear equation was built step-by-step. The basic procedures involve (i) identifying an initial model, (ii) iteratively ‹stepping›, that is repeatedly altering the model at the previous step by adding or removing a predictor variable in accordance with the ‹stepping criteria› (in our case, probability of F = 0.05 for inclusion; probability of F = 0.1 for exclusion for the forward selection method), and (iii) terminating the search when stepping is no longer possible given the stepping criteria, or when a specified maximum number of steps have been reached. Specifically, at each step, all variables are reviewed and evaluated to determine which one will contribute most to the equation. That variable will then be included in the model, and the process starts again. A limitation of the stepwise regression search approach is that it presumes there is a single ‹best› subset of X variables and seeks to identify it. There is often no unique ‹best› subset, and all possible regression models with a similar number of X variables as in the stepwise regression solution should be fitted subsequently to study whether some other subsets of X variables might be better (17, 18). We used GA for variable selection in MLR regression. The applied GA used a binary representation as the coding technique for the given problem: The presence or absence of a descriptor in a chromosome was coded by 1 or 0. The GA performed its optimization by variation and selection via the evaluation of the fitness function. The operators used here were crossover and mutation. The probability for the application of these operators was varied linearly with the generation renewal (0 ± 1% for mutation and 60 ± 90% for crossover). The population size was 100. For a typical run, the evolution of the generation was stopped when 90% of the generations took the same fitness. The fitness function was the root mean square error of cross-validation (19).

*Model validation*

The goodness-of-fit of the resulted QSAR models were judged using statistical parameters such as correlation coefficient (R^2^), standard error of regression (SE), and variance ratio (F) at specified degrees of freedom. The generated QSAR equations were also validated by leave-one-out cross-validation correlation coefficient (Q^2^) and root mean square error of cross-validation (RMScv)(20). In addition, an external test set composed selected11 molecules was used to judge the overall prediction ability of the resulted models (see Table 1). According to Tropsha *et al. *(21). The predictive ability of a QSAR model should be tested on an external set of data that has not been taken into account during the process of developing the model. In particular, to assess the predictive power of QSAR models the correlation coefficient between the predicted and observed activities of compounds from an external test (R ^2^), the correlation coefficients for regressions through the origin (predicted versus observed activities, or observed versus predicted activities, i.e., R_0_^2^ or Rꞌ_0_2, respectively), and the slope of the regression lines through the origin (K and K ‹, respectively) were calculated. Tropsha *et al*. (21, 22). considered a QSAR model to be predictive, if all of the following conditions are satisfied: (i) Q ^2^ > 0.5, (ii) R ^2^ > 0.6, (iii) R_0_^2^ or Rꞌ_0_^2^ is close to R^2^, such that [(R^2^ - R_0_^2^) ⁄ r ^2^] or [(R^2^- Rꞌ_0_^2^) ⁄ R ^2^] < 0.1and (iv) 0.85 ≤ K ≤1.15 or 0.85 ≤ K ‹ ≤ 1.15. In addition, according to the recommendation of Roy and Roy [18], an additional statistic for external validation (rm^2^) was calculated as rm^2^ = R^2^*[1-(R^2^-R_0_^2^)^1 ⁄ 2^]. For a model with good external predictability, rm^2^ value should be > 0.5. (23)

*Y-randomization test*

This technique ensures the robustness of a QSAR model. The dependent variable vector pIC_50_ is randomly shuffled and a new QSAR model is developed using the original independent variable matrix. The new QSAR models (after several repetitions) are expected to have low *R*^2^ and *Q*^2^ values. If the opposite happens then an acceptable QSAR model cannot be obtained for the specific modeling method and data (12).

*Applicability domain*

Finally the QSAR model was used to identify novel active compounds via an in *silico* screening procedure, and thus the definition of its domain of applicability is of particular importance. The utility of a QSAR model is based on its accurate prediction ability for new compounds. A model is valid only within its training domain, and new compounds must be assessed as belonging to the domain before the model is applied. The applicability domain is assessed by the leverage values for each compound. The threshold h* is generally fixed at 3(k + 1) ⁄ n (n is the number of training set compounds, and k is the number of model parameters), On the other hand, when the leverage value of a compound is lower than the threshold value, the probability of accordance between predicted and observed values is as high as that for the training set compounds (24).

*In silico screening*

Finally *in silico* screening procedure was carried out to identify a variety of potential novel lead compounds by presenting structural modifications on the original dataset. Throughout the screening procedure, only the predictions that fall into the domain of applicability were considered reliable.

## Results and Discussion

On the basis of Kennard-Stones algorithm, the dataset of the 46 pyridazine derivatives was divided into a training set (35 compounds) and a prediction or test set (11 compounds, see Table 1). Stepwise regression was used on the training data set to develop MLR QSAR model. 

pIC_50_ = -102.168 (± 15.264) + 119.255(± 17.542) MATS4m + 0.106(± 0.026) RDF 105u – 0.168(± 0.024) RDF100u – 5.458(± 1.131) GATS 3v + 0.269(± 0.053) RDF075v + 1.222(± 0.293) C-005 + 0.073(± 0.022) RDF095u – 0.006(± 0.002) Surface area

N = 35 R^2 ^= 0.837 R^2^_adj_= 0.780 F = 16.079 S.E.= 0.330 Q^2 ^= 0.733

RMScv = 0.374 R^2 ^_pred_= 0.754

The regression coefficients in this model are significant at the 95% level. This equation can predict and explain 75.4% and 83.7%, respectively, of the variance of the inhibitory activity.

**Figure 1:**
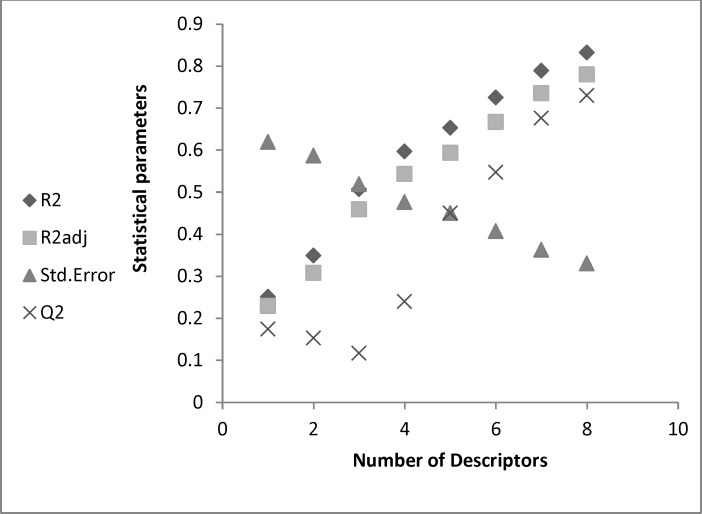
The statistical parameters according to number of descriptors entered in model.

The possibility of having included outliers in our dataset was investigated by calculating the standard residuals. Standardized residuals greater than 2.5 or less than -2*.*5 are considered large and indicate the exclusion of the respective data from the data set. The calculated standardized residuals were within the above upper and lower limits for all the compounds, and thus, none of them were excluded from the data set as outlier (3). The Figure1 showed that the good model was obtained with eight descriptors.

The above equation showed that the most significant descriptors are Moran autocorrelation of lag 4 weighted by mass (MATS4m), Radial Distribution Function - 105 / unweighted (RDF105u), Radial Distribution Function - 100 / unweighted (RDF100u), Geary autocorrelation of lag 3 weighted by van der Waals volume (GATS3v), Radial Distribution Function - 075 / weighted by van der Waals volume (RDF075v), CH3X (C-005), Radial Distribution Function - 095 / unweighted (RDF095u) and surface area. The correlation matrix (Table 2) indicated that the eight selected descriptors are not highly correlated. Variance Inflation Factor (VIF) values for the eight descriptors (25-27), also shown in Table 2, demonstrate that the model contains no multicollinearity.

*Interpretation of the Selected Descriptors*

The variety of factors such as molecular electrostatic potential, polarizability, hydrophobicity, and lipophobicity influence the binding of ligand to its target. MATS4m and GATS3v are Autocorrelation of Topological Structure*. *The 2D-autocorrelation descriptors explain how the values of certain functions, at intervals equal to the *lag*, are correlated. The 2D autocorrelation descriptors represent the topological structure of the compounds, but are more complex in nature when compared to the classical topological descriptors. The computation of these descriptors involves the summations of different autocorrelation functions corresponding to different structural lags and leads to different autocorrelation vectors corresponding to the lengths of the sub-structural fragments. Basically, the pool of 2D autocorrelation descriptors defines a wide 2D space. On behalf of a greater applicability, physicochemical properties (atomic masses, atomic van der Waals volumes, atomic Sanderson electronegativities, and atomic polarizabilities) were inserted as weighting components. As a result, these descriptors address the topology of the structure or parts thereof in association with a specific physicochemical property. Bearing in mind this aspect, the interpretation of 2D autocorrelation descriptors was uneasy. Based on derived model the positive regression coefficient of MATS4m shows that small path lengths and branching in the molecule (lag 4 weighted by atomic mass) contribute to higher activity (28). RDF105u, RDF100u, RDF075v and RDF095u are Radial Distribution Function descriptors; the 3D coordinates of the atoms of molecules can be transformed into a structure code that has a fixed number of descriptors irrespective of the size of a molecule. This task is performed by a structure coding technique referred to as Radial Distribution Function code (RDF code). In general, there are some prerequisites for a structure code:

- Independent from the number of atoms, i.e., the size of a molecule,
- Unambiguity regarding the three-dimensional arrangement of the atoms, and
- Invariance against translation and rotation of the entire molecule.

Formally, the Radial Distribution Function of an ensemble of N atoms can be interpreted as the probability distribution to find an atom in a spherical volume of radius r. The equation represents the Radial Distribution Function code as it is used in this investigation:

$g\left(r\right)=f\times \sum_{i}N-1\sum_{i}N{\mathrm{AiAje}}^{{-B(r-\mathrm{ri}j)}^{2}}$

Where f is a scaling factor and N is the number of atoms. By including characteristic atomic properties A of the atoms i and j, the RDF codes can be used in different tasks to fit the requirements of the information to be represented. 

The exponential term contains the distance rij between the atoms i and j and the smoothing parameter B, which defines the probability distribution of the individual distances. g(r) was calculated at a number of discrete points with defined intervals The radial distribution function in this form meets all the requirements for 3D structure descriptors: it is independent of the number of atoms, i. e., the size of a molecule, it is unique regarding the three-dimensional arrangement of the atoms, and it is invariant against translation and rotation of the entire molecule. Additionally, the RDF descriptors can be restricted to specific atom types or distance ranges to represent specific information in a certain three-dimensional structure space, e.g. to describe steric hindrance or structure/activity properties of a molecule. Finally, the RDF descriptors are interpretable by using simple rules sets, and thus it provides a possibility for conversion of the code back into the corresponding 3D structure. Besides information about interatomic distances in the entire molecule, the RDF descriptors provide further valuable information, e.g. about bond distances, ring types, planar and non-planar systems and atom types. By using different weighting schemes, which include atom types, electronegativity, atom mass or van der Waals radii, RDF can be adjusted to select among those atoms of molecule, which give rise to an important descriptor in deriving an appropriate QSAR (29-31). 

Final descriptor C005 is one of the Ghos–Crippen atom-centred fragments related to the methyl group attached to any electronegative atom (O, N, S, P, Se, halogens) fragment. It gives information about the number of predefined structural features in the molecule. Based on the produced QSAR equation a high value of MATS4m, RDF105u, RDF075v, C-005 and RDF095u give a positive contribution to the IL-1 production inhibition. On the other hand, a high value of GATS 3v and Surface area give a negative contribution to the inhibition.

*Model Validation*

The IL-1β production inhibition predictability of the proposed model was evaluated by using the external set of 11 compounds (Table 1). The proposed QSAR model has all conditions to be considered as predictive models.

R^2 ^_pred = _0.754 > 0.6

[(R^2^_pred_ - R_0_^2^) ⁄ R^2^_pred_] = -0.078 < 0.1

[(R^2^_pred_ – R׳_0_^2^) ⁄ r^2^_pred_] = -0.061<0.1

rm^2 ^= 0.595> 0.5

K= 0.83, K׳=1.00

This model was further validated by applying the Y-randomization test. Several random shuffles of the Y vector were performed. The low *R*^2^ (0.0 <* R*^2 ^< 0.34) and *Q*^2 ^(0.0 < *Q*^2 ^< 0.25) values indicate that the good results in our original model are not due to a chance correlation or structural dependency of the training set. The extrapolation method was applied to the compounds that constitute the test set. The results are presented in Table 1. None of the 11 compounds fell outside from the domain of the model (warning leverage limit = 0.77).

The suggested method, according to the high predictive ability, could be a useful tool to the costly and time consuming experiments for determining the IL-1 β production inhibitory activity of pyridazine derivatives. The method can also be used to the screen virtual compounds in order to identify derivatives with desired activity.

*In silico screening*

The *in silico *screening was applied to the design of new structures with potential IL-1 β production inhibitors according to the developed QSAR model. The role of the *in silico* screen was as a guide to the identification of the most promising new synthetic targets. For this purpose, first we selected potent inhibitors from data set (3, 4, 17, 22, 23, 24, 28, 31, 32, 33, 34, 35, 36, 37) and retain the main scaffold (4-methoxy phenyl moiety) and change the heterocyclic core, aromatic moiety and connection between aromatic moiety and central heterocyclic core. The modifications incorporated in the virtual screening study were chosen based on their synthetic feasibility. The suggested structures were not involving the use of unusual ring fragments or functional groups that cannot be prepared using established protocols. The chemistry of 1,2,4-triazine was well understood and introducing practical modifications here was considered synthetically viable, the *in silico* screen began with the replacement of the pyridazine core by 1,2,4-triazine (Table 3). The model tolerated the introduction of 1,2,4-triazine since all those studied were within the domain of applicability. The compounds 49 and 51 showed the best activity, 6.83 and 6.56, respectively. In some cases the activities lower and just one compound has higher activity than original compound. The next step was incorporated 1,2,4-triazine instead of pyridazine core in second series of compounds that the different substituted aromatic moiety connect to central heterocyclic by O or S atoms. This model tolerated and the activity was retained (Table 4.). 

**Table 1:** IL-1 β production inhibitory activity—observed and predicted using developed model

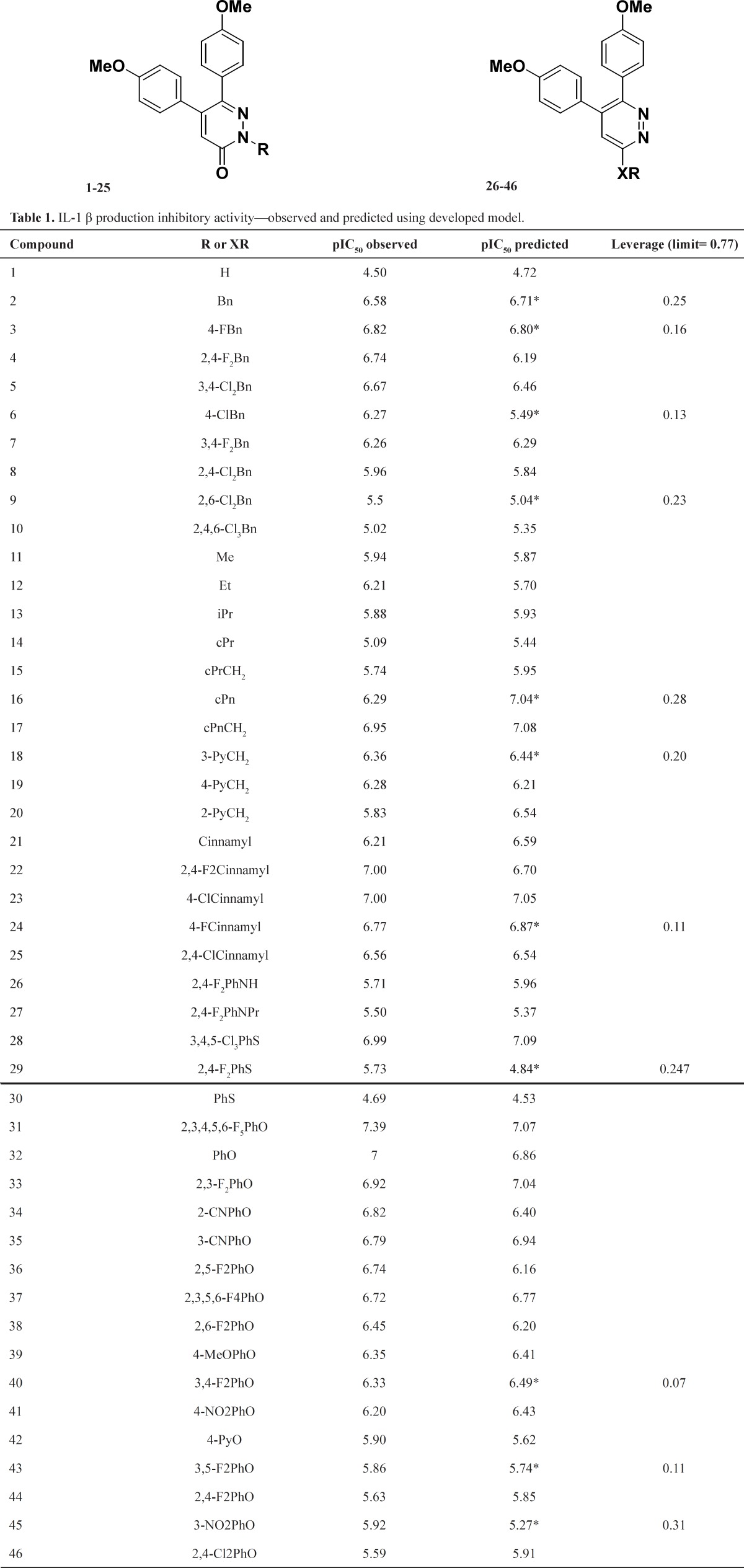

*Selected as test set.

**Table 2:** Correlation matrix for the eight selected descriptors

	MATS4m	GATS3v	RDF095u	RDF100u	RDF105u	RDF075v	C-005	Surface area	VIF*
MATS4m	1	0.598	0.185	0.244	0.129	0.307	-0.273	0.480	3.785
GATS3v		1	0.064	0.143	0.242	0.379	-.005	0.214	3.143
RDF095u			1	0.612	0.511	0.235	-0.154	0.548	2.830
RDF100u				1	0.608	0.555	-0.209	0.614	2.719
RDF105u					1	0.639	-.099	0.585	2.744
RDF075v						1	-0.305	0.485	1.478
C-005							1	-0.132	2.389
Surface area								1	3.563

* VIF less than 10 demonstrates that the model contains no multicollinearity.

**Table 3:** Predicted activities of selected 1,2,4-triazin-3(2H)-one derivatives

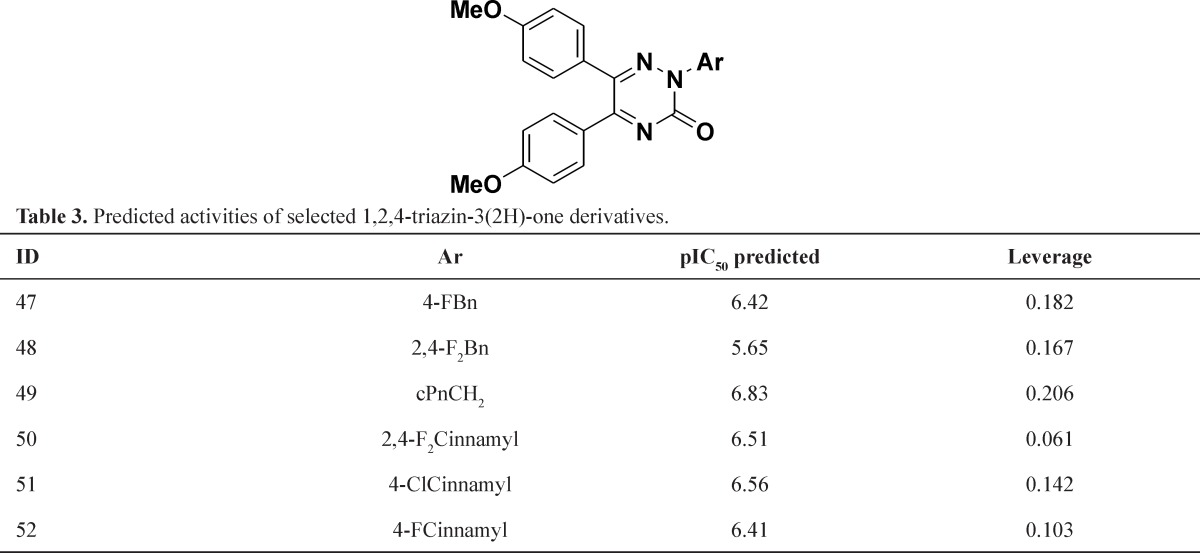

**Table 4.** Predicted activities of selected 3-aryl 1,2,4-triazin derivatives

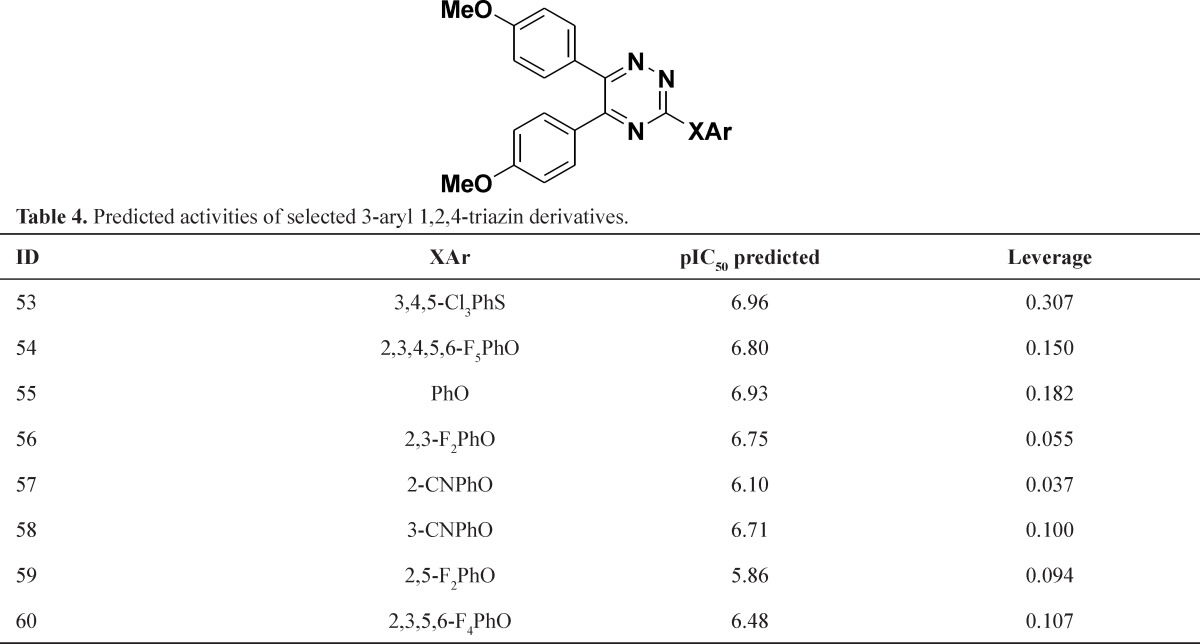

**Table 5:** Predicted activities of selected 3-amino 1,2,4-triazin derivatives

ID	XAr	pIC_50_ predicted	Leverage
61	3,4,5-Cl_3_PhNH	7.51	0.350
62	2,3,4,5,6-F_5_PhNH	6.67	0.120
63	PhNH	6.89	0.182
64	2,3-F_2_PhNH	6.72	0.055
65	2-CNPhNH	6.29	0.034
66	3-CNPhNH	6.90	0.101
67	2,5-F_2_PhNH	5.84	0.099
68	2,3,5,6-F_4_PhNH	6.36	0.103

**Table 6:** Predicted activities of selected 3-benzylidenehydrazine 1,2,4-triazin derivatives

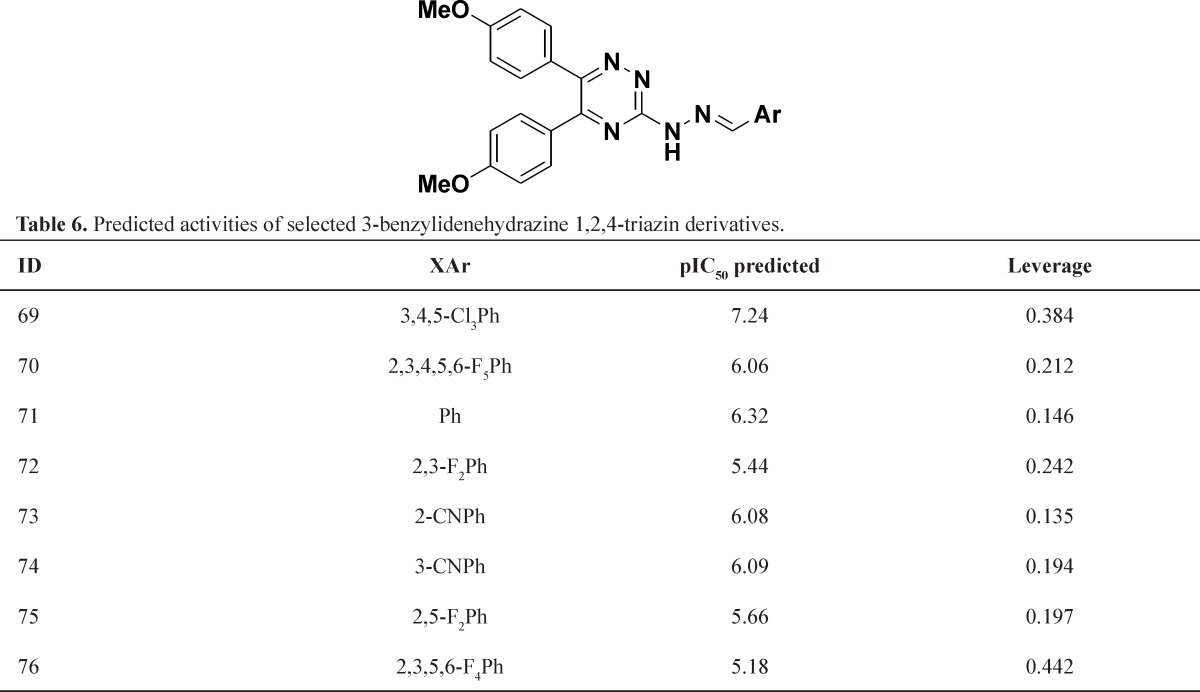

**Table 7:** Predicted activities of selected three aryl pyridine derivatives

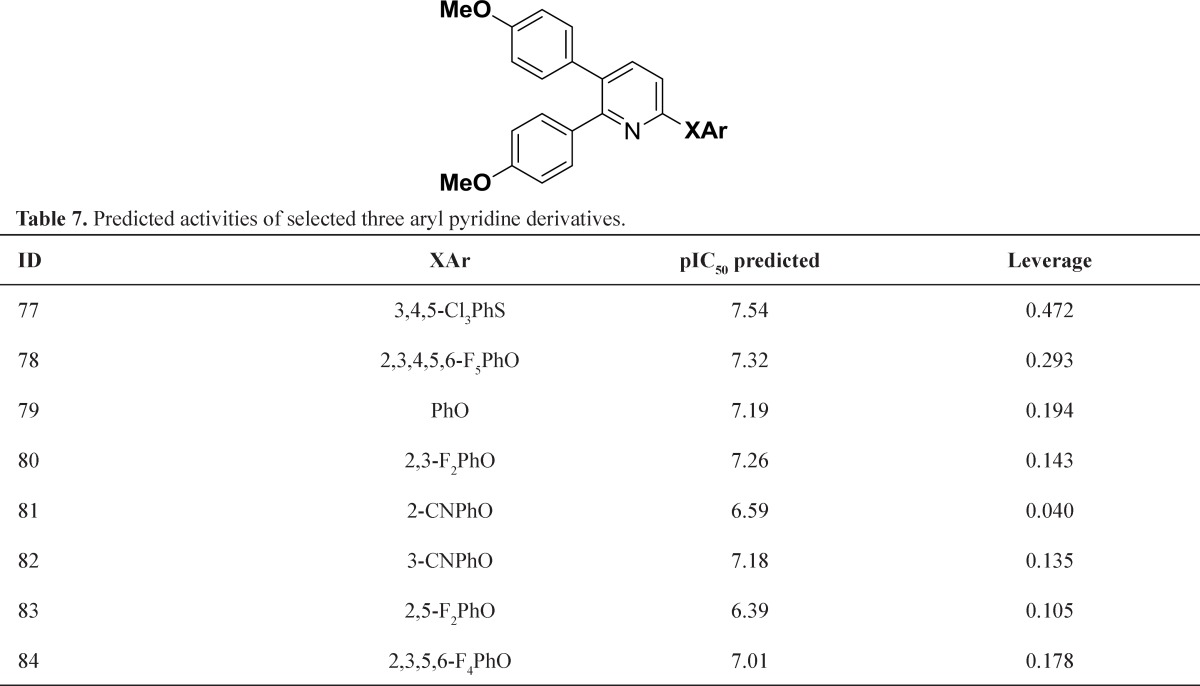

The other modification was replacement connective atoms (S or O) to N, this modification gave structures that showed good activity and were within the models domain of applicability (Table 5) but introducing the second nitrogen to linker slightly lead to decrease of predicted activity and was clearly within the domain of applicability (Table 6). Interestingly, replacement of pyridazine to pyridine ring led to enhanced biological activity and comfortably within the domain of applicability (Table 7). The *In silico* study suggested that presence of heterocyclic ring containing nitrogen group was necessary for inhibition Il-1 β production and increasing number of nitrogen ring diminished 

biological activity.

## Conclusion

In this study we have identified eight descriptors that successfully model the IL-1 β production inhibitory activity. The validation procedures utilized in this work (separation of data into independent training and validation sets, Y-randomization) illustrated the accuracy of produced QSAR model. Based on the developed QSAR model, we have designed novel structures that could be further investigated as novel effective IL-1 beta production inhibitors. The proposed method, due to the high predictive ability, tenders a useful alternative to the costly and time consuming experiments for cytokine production inhibitory activity.
